# A tracheal ‘tumor’ revealed as granulation-encapsulated sunflower seed shell in a 23-month-old child

**DOI:** 10.1016/j.bjorl.2026.101781

**Published:** 2026-03-27

**Authors:** Xiaowei Chen, Feizhou Zhang, Jia Liu, Qunying Li, Yue Jin, Lei Wu

**Affiliations:** a Zhejiang University School of Medicine, The Children’s Hospital, Department of Otorhinolaryngology, Zhejiang, China; b National Clinical Research Center for Children and Adolescents’ Health and Diseases, China; c Zhejiang University School of Medicine, The Children’s Hospital, Department of Pulmonology, Zhejiang, China; d Zhejiang University School of Medicine, The Children’s Hospital, Department of Endoscopy Center, Zhejiang, China; e Zhejiang University School of Medicine, The Children’s Hospital, Department of Radiology, Zhejiang, China; f Zhejiang University School of Medicine, The Children’s Hospital, Department of Anesthesiology, Zhejiang, China

## Introduction

Pediatric bronchial foreign bodies are a highly dangerous emergency in clinical pediatric practice, predominantly affecting children under 5-years of age, particularly infants and toddlers under 3-years-old.^1^ Due to their immature masticatory function, underdeveloped laryngeal protective reflexes, and the common tendency to grasp and put small objects in their mouths, children are at risk of inhaling plant-based foreign bodies such as peanuts, melon seeds, and beans, as well as non-plant-based items like toy parts and coins, which can easily pass through the glottis and lodge in the bronchus. This obstruction can provoke acute symptoms such as severe coughing, wheezing, and difficulty breathing. If not recognized and treated promptly, it may lead to severe complications including pneumonia, atelectasis, and emphysema, and can even pose a life-threatening risk due to asphyxia. Therefore, early recognition of the history of foreign body inhalation, along with imaging studies (such as chest X-Ray, computed tomography, or bronchoscopy) for accurate diagnosis, followed by urgent removal of the foreign body via bronchoscopy, is crucial for reducing both mortality and the risk of long-term complications in affected children.^2^

Herein, we report a case in which a neck and chest computed tomography angiography raised suspicion of a tracheal mass. Surgical exploration revealed a foreign body encased in granulation tissue. This emphasizes the precise collaboration between flexible and rigid bronchoscopy, conducted under safe anesthesia conditions, as the gold standard for diagnosing airway foreign bodies.

## Case presentation

**Fig. 1:**
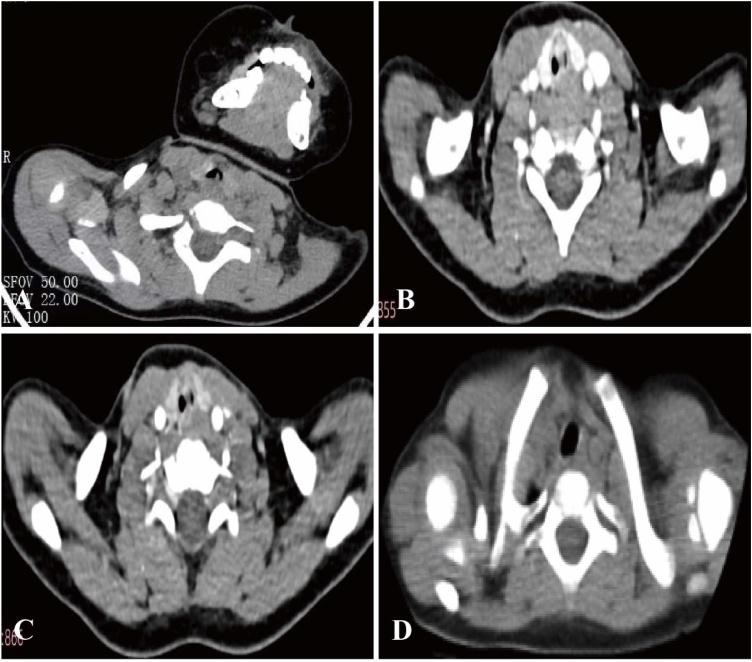
Chest computed tomography and computed tomography angiography images. (A) A nodular high-density lesion is noted at the subglottic-tracheal junction. (B) Venous phase: A nodular high-density lesion along the medial wall of the left trachea (at thyroid gland level) demonstrates mild density inhomogeneity. (C) Arterial phase: The lesion shows marked enhancement. (D) No evident mass lesion is identified within the trachea.

A previously healthy 23-month-old male infant was admitted to our hospital on March 18, 2025 with stridor and hoarseness persisting for over 10-days. His parents initially denied a history of foreign body aspiration. The patient had received 5-day inpatient care at a local hospital from March 13 to 17, 2025, including nasal cannula oxygen therapy, intravenous azithromycin (10 mg/kg/day) for empiric antimicrobial coverage, and intravenous methylprednisolone sodium succinate (2 mg/kg q12 h) for anti-inflammatory and bronchodilatory effects, but no significant clinical improvement was observed. A chest computed tomography scan at the local hospital revealed a subglottic nodular lesion at the tracheal junction (Fig. 1A), prompting transfer to our facility for multidisciplinary airway evaluation and intervention.

On admission, the patient’s vital signs included height 82 cm, weight 11 kg, temperature 36.6 °C, blood pressure 100/60 mm/Hg, heart rate 102 beats/min, respiratory rate 26 breaths/min, and room air oxygen saturation 96%. Inspiratory tracheal tug was positive and inspiratory wheezes were auscultated bilaterally. No peripheral edema, rash, or abnormalities were found on cardiovascular and abdominal examinations.

After admission, the patient underwent fiberoptic laryngoscopy and nasopharyngoscopy, both of which revealed no significant abnormalities. A neck and chest computed tomography angiography performed on March 19, 2025 for tumor assessment indicated a soft tissue mass on the left wall of the trachea at the thyroid level (Fig. 1B). Routine laboratory tests including respiratory pathogen screening, complete blood count with high-sensitivity C-reactive protein, biochemical profile, coagulation profile, allergen testing with immunoglobulins and complement, stool and urine routine, blood gas analysis with electrolytes, lactate and glucose, and sputum culture showed no significant abnormalities.

Given the complex condition, a multidisciplinary consultation was organized involving experts from pulmonology, otorhinolaryngology, radiology, endoscopy center, anesthesiology, and the medical affairs department. After comprehensive analysis of the patient’s medical history, examination results, and clinical features, the probable diagnosis of a tracheal tumor was confirmed, and surgical exploration was deemed necessary. Written informed consent was obtained from the parents after detailed explanation of the surgical requirements and risks.

**Fig. 2:**
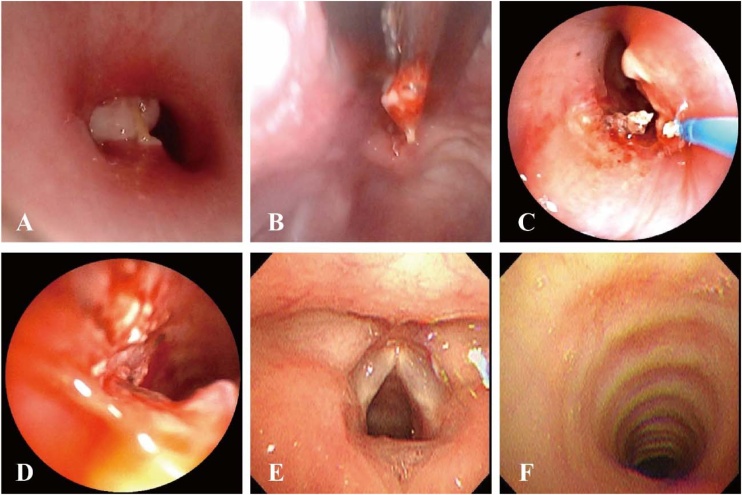
Bronchoscopic findings. (A) A white reflective foreign body was visualized at the subglottic-tracheal junction, obstructing the tracheal lumen. (B) A sunflower seed shell encased in granulation tissue was successfully extracted via rigid bronchoscopy. (C) Laser cauterization of residual granulation tissue was performed under flexible bronchoscopy. (D) Post-procedural assessment revealed significant improvement in tracheal patency following laser cauterization. (E) Follow-up flexible bronchoscopy demonstrated no evidence of glottic injury. (F) Subsequent flexible bronchoscopy showed absent tracheal scar formation along the mucosal margin and near-complete resolution of granulation tissue.

**Fig. 3:**
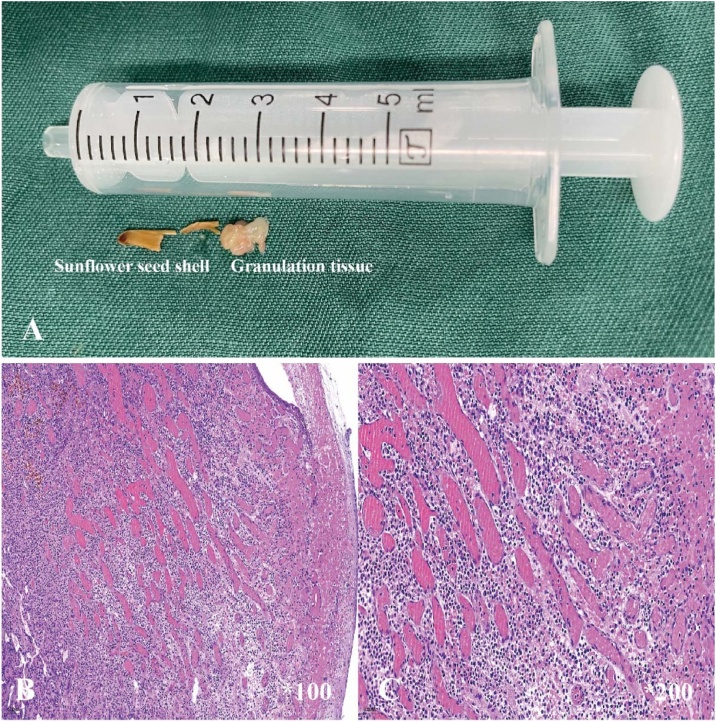
The foreign body and histopathological examination. (A) Tracheal foreign body: A sunflower seed shell encased in granulation tissue. (B) Hematoxylin-eosin staining (100× magnification): Inflammatory cell infiltration was observed. (C) Hematoxylin-eosin staining (200× magnification): No tumor cells were identified, with only inflammatory cells present.

On March 21, 2025, the patient was transferred to the operating room for the planned procedure. Under low-dose intravenous general anesthesia, a fiberoptic bronchoscope was inserted through the nose. A suspicious foreign body surrounded by extensive granulation tissue was identified in the trachea below the glottis (Fig. 2A). Examination of the upper esophagus showed no significant mucosal damage. The fiberoptic bronchoscope was then removed and a rigid bronchoscope was used orally to successfully retrieve the foreign body, which was identified as a sunflower seed shell approximately 2 cm in length (Fig. 3A).

Under laryngeal mask anesthesia, a fiberoptic bronchoscope was reinserted to examine the airway. Significant residual granulation tissue around the former foreign body site was observed, causing marked tracheal stenosis and persistent inspiratory tracheal retraction. The surgical team meticulously removed the granulation tissue using endoscopic snares, lasers, and biopsy forceps (Fig. 2B‒D) until airway patency was significantly improved and inspiratory tracheal retraction was alleviated. Deep airway blood was washed out and further examination of the lower airway revealed no additional foreign bodies or tumors. The entire surgical procedure proceeded smoothly with stable vital signs throughout.

Postoperatively, the patient was transferred to the intensive care unit for close monitoring. When intraoperative findings were communicated to the family, they recalled the child had eaten sunflower seeds over a month prior. The patient’s condition remained stable with normal vital signs and no apparent discomfort. Histopathological examination of the granulation tissue showed reactive granuloma and infiltration of various inflammatory cells (Fig. 3B and C). A follow-up fiberoptic bronchoscopy one week later showed no obvious residual granulation tissue in the trachea (Fig. 2E).

## Discussion

Pediatric foreign body aspiration refers to objects accidentally inhaled and lodged in the larynx, trachea, or bronchi of children, causing partial or complete airway obstruction. This condition manifests as respiratory distress, hypoxia, or life-threatening asphyxia. History of foreign body aspiration is crucial for diagnosis. Preoperatively, despite repeated inquiries about a history of foreign body aspiration, the family denied it. However, intraoperatively, the mass found was a sunflower seed shell wrapped in granulation tissue. Then the family recalled the child had eaten sunflower seeds over a month ago.

Plant-based items (peanuts, seeds) and non-organic materials (button batteries, toy parts) are common objects in foreign body aspiration. High-risk scenarios include eating while crying, running, or playing. The aspirated foreign body is a sharp-edged sunflower seed shell which can scratch the airway mucosa and cause airway inflammation. Over a month after the foreign body aspiration, the foreign body triggered granulation tissue growth leading to airway obstruction and the child subsequently developed stridor and hoarseness that persisted for over 10-days. Prolonged retention of foreign bodies in the respiratory tract can lead to more severe complications so early recognition becomes even more important.^3^

Imaging like chest X-Rays or computed tomography scans helps locate the foreign body. Chest computed tomography showed a nodule at the subglottic area and tracheal junction. Fiberoptic laryngoscopy and nasopharyngoscopy revealed no obvious abnormalities. A computed tomography angiography of the neck and chest indicated a tracheal soft tissue mass, about 5.4 × 4.7 × 5.4 mm in size, with uneven density and a computed tomography value of about 57 Hounsfield Units (the standard metric for quantifying tissue density in computed tomography imaging). After contrast agent injection, the lesion enhanced significantly, with computed tomography values of about 92 Hounsfield Units, 149 Hounsfield Units, and 157 Hounsfield Units in the three phases. This progressive enhancement pattern aligns with the typical vascularity of pediatric airway tumors that feature abundant neovascularization to promote contrast agent uptake and retention and helps distinguish it from non-neoplastic lesions such as simple granulation tissue or cysts, which usually show mild or no enhancement. Combined with the mass-like morphology on computed tomography angiography, this radiological feature strongly suggested an airway tumor. However, the child was finally diagnosed via bronchoscopy with a tracheal foreign body encased in granulation tissue, which indicates that imaging cannot serve as the gold standard for diagnosing respiratory foreign bodies. In clinical practice, we have encountered bronchial foreign bodies that were radiologically negative,^4^ which suggests that we should not over-rely on radiological signs.

Prompt removal of the foreign body is crucial. Commonly, bronchoscopy a minimally invasive procedure is used. For large or unusually located foreign bodies, tracheotomy or thoracoscopy may be necessary. In emergencies, the Heimlich maneuver can relieve airway obstruction. The child was an infant with a soft tissue mass in the upper trachea, leading to extremely high anesthesia risks. After thorough communication with the parents and informing them of the relevant risks, we conducted a surgical exploration in the operating room. An otolaryngologist was on standby for a tracheotomy, and after general anesthesia, a fiberoptic bronchoscopy was performed first. A suspicious foreign body was found. Under laryngeal mask anesthesia, we successfully removed a 2-cm-long sunflower seed shell surrounded by granulation tissue at the tip using a rigid bronchoscope. Due to the child’s significant airway obstruction symptoms, we cleared the granulation tissue using a snare, laser, and biopsy forceps under fiberoptic bronchoscopy. In clinical practice, we have found that a long-standing undiagnosed airway foreign body can lead to inflammatory granulation tissue formation, causing airway stenosis or even complete blockage. Secondary bacterial infection may result in necrotizing pneumonia. Airway intervention is needed for these severe complications.^5^

## Conclusion

History of foreign body aspiration is key to diagnosis. Long-standing foreign bodies with granulation tissue may mimic respiratory tumors. Successful removal depends on multidisciplinary collaboration among Anesthesiology, Pulmonology, and Otorhinolaryngology.

## ORCID ID

Xiaowei Chen: 0000-0001-7425-8715

Feizhou Zhang: 0000-0002-2377-248X

Jia Liu: 0000-0001-7198-0956

Qunying Li: 0009-0005-0437-9403

Yue Jin: 0009-0004-1021-910X

## CRediT authorship contribution statement

CXW and ZFZ completed the first draft, LJ, LQY, JY have participated to the data collection and improved the later revision of the article, WL revised the manuscript to ensure the authenticity and practicability. All authors approved the final manuscript as submitted and agree to be accountable for all aspects of the work.

## Consent for publication

These children's parents have informed and consented to the publication.

## Ethics approval and consent to participate

This study was approved by the ethics committee of the children's hospital affiliated to Zhejiang university school of medicine and informed consent was obtained from these children's parents.

## Funding

There is no funding support for the work.

## Clinical trial number

Not applicable.

## Data availability statement

The authors declare that all data are available in repository.

## Availability of data and materials

All data generated or analyzed during this study are included in this published article (and its supplementary information files).

## Declaration of competing interest

The authors declare no conflicts of interest.
